# Modus Operandi and Domain of the Turkish Bath

**Published:** 1870-02

**Authors:** M. P. Hanson

**Affiliations:** Milwaukee, Wisconsin


ARTICLE II. — *Modus Operandi and Domain of the Turkish Bath.* By M. P. HANSON, M.D., Milwaukee, Wisconsin.

PRELIMINARY to a perfect understanding of principles and facts which I propose to set forth in this treatise, it is necessary to state very briefly the *modus operandi* of the thermæ.

One of the uses of ordinary perspiration is to maintain the equilibrium of temperature, on which the safety of the individual depends. The natural average temperature of the human body is 98° Fahr., and whenever it rises to 112°, or sinks to 60°, death ensues. Therefore nature has provided that this standard of 98° shall be maintained in every latitude, and under all atmospheric variations. When unusual heat is applied to the surface of the body, the system is called upon to protect itself by maintaining its equilibrium at 98°, and this is accomplished by throwing out water to be evaporated from the surface. When water becomes vapor, a large amount of heat becomes latent, and thus the higher the temperature, the more abundant the flow of sweat, and the more rapid the evaporation, and the heat is carried off latent in the vapor. Thus man is enabled to endure habitual exposure to an artificial temperature of dry air, as high as 400°. Carpenter says:

"Many instances are on record of heat from 250° to 280°, being endured in dry air, for a considerable length of time, even by persons unaccustomed to a particularly high temperature; and persons whose occupations are such as to require it, can sustain a much higher degree of heat, though perhaps for not any long period. The workmen of the late Sir F. Chantrey have been accustomed to enter a furnace in which his moulds were dried, whilst the floor was red hot, and a thermometer in the air stood at 360°; and Chabert, 'the Fire King,' was in the habit of entering an oven, whose temperature was from 400° to 600°." — *Human Physiology, Third English Edition, p.* 888.

Men engaged in various branches of manufacturing industry, such as ore smelting, iron foundries, rolling mills, and glass works, are habitually exposed, for lengthened periods, to heat, ranging from 200° to 350°, which they are able to sustain for years, without any loss of vigor, or detriment to health, and simply because there are seven millions little glands spread over the inner surface of the skin for the purpose of secreting an aquæous fluid for exhalation and evaporation; thus carrying off the heat that would otherwise raise the temperature of the body. Water boils at 212°, when steam is formed, and not all the coal in Pennsylvania, heaped into one monstrous fire, could raise the temperature of water above 212°, so long as the steam or vapor is allowed to pass off. And just so a man can endure a temperature of even 600°, for a time, with impunity, provided the skin acts freely, and the atmosphere about him is *dry*, and drinks up rapidly the moisture that exhales, as dry air always will. So far, indeed, from high temperature proving injurious, it is owing to the adaptability of the skin to resist high artificial heat, that the bath relies for its marvelous sanitary and curative powers; and thus we see that there is no foundation for the popular, and, to some extent, professional prejudice about the hot-air bath increasing the temperature of the body to a dangerous degree. Such a prejudice is not only condemned by physiology and experience, but is in *direct antagonism to the very laws of our being.*

Now, how comes it that by a slight increasing of heat, there should be a sudden power given to the skin to throw out moisture? And where does this moisture come from? It comes from the blood; and to be able to furnish the moisture, the *skin must have blood;* and this is the grand secret of the *modus operandi* of the Turkish bath. *It is by equalizing the circulation*, and *thus removing congestion*, *whether of the lungs*, *the liver*, *the brain*, *or any internal organ*, *or tissue near the surface*, *or the skin itself.* When the heat is applied to the whole surface of the body, the blood that is freely circulating about the trunk is thrown to the surface, and the extremities; and its water, loaded with the effete and waste matter from the system, is given up to protect the skin; but the heat is continued, and more water is wanted, and more blood must be had, and now all the energies of the system are aroused to bring into the circulation the pounds of blood that have been stagnant, and congesting your lungs, or liver, or some other organ, for weeks, and all the absorbents are set to work, to take up any dropsical effusion, whether in the cellular tissue, or in the shut cavities, as the abdomen, chest, etc., that it may be restored to the circulation, and evaporated from the surface.

So, also, in equalizing the circulation, it distributes nutrition, as nutrition goes with the blood. Erasmus Wilson says: " Scrofula is imperfect nutrition; cancer is imperfect nutrition; indigestion, rheumatism, gout, neuralgia, are imperfect nutrition. Give a power by which nutrition can be regulated, and you can immeditely control these various diseases. Now there is no power by which the proper direction of nutrition can be attained, excepting through the skin, and that I believe to be the explanation of the extraordinary results which flow from the Turkish bath."

And, over and above all, the bath *purifies the blood* more directly and thoroughly than any other means the science of medicine has ever before suggested. Perspiration eliminates the water from the blood, loaded with the waste and poisonous matter from the system; and, by drinking cold water, the blood is replenished with wholesome material in exchange. Indeed, this is the most important purpose which perspiration serves. Man perspires, because perspiration, being the watery portion of the blood, carries out with it all extraneous and poisonous matter; thus removing all constitutional taints, whether of scrofula, syphilis, cancer, tuberculosis, or any blood disease.

The blood has to carry away the worn-out material which results from every motion of the body, or of the mind. While depositing fresh fibre, it has to remove old poison. In fact, that blood, with its watery portion, is washing the whole internal man, every instant of time, or, he is repeating back upon himself his own pollution.

While, then, we are constantly producing the phenomena of life, we are constantly producing the elements of death. Your own breath will kill; all that comes from you kills; it is poison, and the simple action of the thermæ, or Turkish bath, is to

Equalize the circulation,

Remove congestion,

Distribute nutrition,

Restore and keep active all the secretions, whether of the skin, the kidneys, or the liver, and thus eliminate poison from the system.

And any experienced physician will be able to see how many diseases may be benefited, or entirely controlled, by such an instrumentality.

The Turkish bath, however, in the minds of even medical men, is very generally confounded with the Russian, or steam bath, while neither of them is very well known to the profession. In structure and manipulation and general management, they are essentially the same; neither is represented by a tub or a steam-box, but should consist, at least, of four good-sized rooms. And right here the difference, which is vast and important, begins. The Turkish bath is for the application of dry, heated air (of sufficient temperature) to the surface of the body, and for sufficient time to produce profuse perspiration. On the other hand, the Russian bath is for the application of steam or vapor; and no scientific man, upon a little reflection, can fail to see the difference.

Water at 110° is quite scalding, and vapor at 136° can be endured for only a very short period, because there is no *evaporation* to protect the skin; no heat becomes latent, as in the process of evaporating the perspiration in the hot air bath.

The temperature of the human body is 98°. Immerse it in vapor at 130°, and, it being the coldest object in the room, becomes the condenser, and the water (not the sweat) begins to run from the body, which condenses it; and it is right here that *science* and *experience* meet. The pulse begins to rise, the face is suffused, the head is bewildered, and the sensible heat of the body increased towards the fatal degree. On the contrary, the Turkish bath is only comfortable at 140°, and is used with perfect safety at 240°. At 140° it does not increase the pulse four beats in a minute, while the water bath at 110°, and the Russian bath at 130°, will increase the pulse forty to fifty beats a minute, and cannot be endured more than one hour, while patients frequently remain in the bath of Dr. Urquhart, at Riverside, London, for six hours, and a part of the time at 230°; and, so far from being injured, the happiest results are thus secured.

Another essential difference is, that prolonged bathing in vapor, or water, produces great debility, as it *hinders* perspiration and robs the body of its electricity. Nature never does a work of supererogation; if the moisture is supplied from without, it does not come from within. In the *warm* water bath the parts under water do not perspire, and the patient increases in weight by absorbing the water. In the *hot* water, or vapor bath, he neither increases or diminishes in weight, because the irritation shuts up the pores, in order to protect the tissues beneath. *Hot air*, on the contrary forces a free action of the skin, and, from its dryness, is highly electric. Hence the *invigorating* effects of the one, and the contrary effects of the other.

[TO BE CONTINUED.]

